# Microbiome Profiles of Commercial Broilers Through Evisceration and Immersion Chilling During Poultry Slaughter and the Identification of Potential Indicator Microorganisms

**DOI:** 10.3389/fmicb.2018.00345

**Published:** 2018-03-02

**Authors:** John A. Handley, Si Hong Park, Sun Ae Kim, Steven C. Ricke

**Affiliations:** Center for Food Safety, Department of Food Science, University of Arkansas, Fayetteville, AR, United States

**Keywords:** poultry, *Salmonella*, *Pseudomonas*, microbiome, next generation sequencing, slaughter

## Abstract

Commercial poultry abattoirs were evaluated to determine the efficacy of the multi-hurdle antimicrobial strategy employed to reduce the microbial load present on incoming broilers from the farm. As next generation sequencing (NGS) has been recently employed to characterize the poultry production system, this study utilized 16S High throughput sequencing (HTS) and quantitative plating data to profile the microbiota of chicken carcasses and determine the efficacy of the multi-hurdle antimicrobial system. Aerobic plate count (APC) and *Enterobacteriaceae* (EB) microbial counts were quantified from whole bird carcass rinsates (WBCR). The remaining rinsates underwent microbiome analysis using 16S rRNA gene fragments on an Illumina MiSeq and were analyzed by Quantitative Insights into Microbial Ecology (QIIME). The key stages of processing were determined to be at rehang, pre-chill, and post-chill as per the *Salmonella* Reduction Regulation (75 Fed. Reg. 27288–27294). The APC microbial data from rehang, pre-chill, and post-chill were mean log 4.63 CFU/mL, 3.21 CFU/mL, and 0.89 CFU/mL and EB counts were mean log 2.99 CFU/mL, 1.95 CFU/mL, and 0.35 CFU/mL. NGS of WBCR identified 222 Operational Taxonomic Units’ (OTU’s) of which only 23 OTU’s or 10% of the population was recovered post-chill. Microbiome data suggested a high relative abundance of *Pseudomonas* at post-chill. Additionally, *Pseudomonas, Enterobacteriaceae*, and *Weeksellaceae Chryseobacterium* have been identified as potential indicator organisms having been isolated from all processing abattoirs and sampling locations. This study provides insight into the microbiota of commercial broilers during poultry processing.

## Introduction

The meat processing industry is subject to many regulatory requirements due to the association of foodborne illness outbreaks in which *Salmonella* spp. has been the etiological agent in an estimated 1.0 million food borne illness cases (Scallan et al., 2011; 75 Fed. Reg. 27288–27294). Regulatory requirements established in 1996, set forth by the United States Department of Agriculture Food Safety Inspection Service (USDA-FSIS), required broiler processors to implement both a Hazard Analysis Critical Control Point System (HACCP) and to comply with performance standards for *Salmonella* spp. and *Escherichia coli* Biotype I (Food Safety and Inspection Service USDA, 1996a,b,c). The Food Safety and Inspection Service USDA (2010), introduced modifications to the regulation that both updated existing performance standards and added *Campylobacter* spp. performance standards for broilers (75 Fed. Reg. 27288–27294). Within the Code of Federal Regulations, 9 CFR 381.94 (Food Safety and Inspection Service USDA, 1996b), poultry abattoirs are to test carcasses to demonstrate process control. Additionally, the HACCP plan must be validated annually per 9 CFR 417 (Food Safety and Inspection Service USDA, 1996a) and interventions are a part of the HACCP plan.

Aside from the regulatory requirments, monitoring the microbial intervention will ensure optimal performance in reducing the bacterial load from live hang to post-chill (Stopforth et al., 2007). Broilers brought into the slaughterhouse have been recorded as having aerobic bacterial levels ranging from mean log_10_ 6 to 9 CFU/mL or 4 × 10^8^ to 4 × 10^11^ CFU/carcass (Lillard, 1989, 1990; Kotula and Pandya, 1995). In order to reduce the microbial load effectively, research efforts have focused on the reduction and elimination of both pathogenic and spoilage bacteria (Mulder et al., 1987; Izat et al., 1990; Dickens et al., 1994; Lillard, 1994; Doyle and Waldroup, 1996; Yang et al., 1998; Stopforth et al., 2007; Bauermeister et al., 2008; Northcutt et al., 2008; Millilo et al., 2011; Purnell et al., 2014; Kim et al., 2017). Therefore, antimicrobials commonly undergo evaluations by researchers, both academic and industry, to investigate the efficacy and for improvements to the current system. A method to measure the intervention process is to perform bio-mapping.

Bio-mapping measures the microbial recovery pre- and post-intervention for the whole process. Thus, a systematic analysis of each individual hurdle comprising the whole system. This map will effectively reveal where intervention strategies are successful or failing. In order to measure the effectiveness of commercial intervention strategies against potential pathogens, the employment of indicator organism can prove useful (Russell, 2000; Whyte et al., 2004; James et al., 2006; Handley et al., 2015; Kim et al., 2017). For instance, Enterobacteriaceae is a family of bacteria that contains pathogens such *E. coli* O157:H7 and *Salmonella* spp. (Whyte et al., 2004). An indicator organism would ideally be a non-pathogenic microorganism that behaves similarly to the environmental conditions as a target human pathogen and the population present in large enough quantities to be detected using cost effective microbiological techniques.

Carcasses entering the abattoir yield high levels of bacteria capable of degrading the product quality and/or causing human pathogenesis (Lillard, 1989, 1990; Kotula and Pandya, 1995; Stopforth et al., 2007). The identified microbiota present through various stages of food processing should enable researchers and industry experts to better develop product and intervention strategies (Solow, 1993; Stern et al., 2001; Hunter et al., 2009). The bacterial populations that are present can be indicative of contamination or it may be inherent to the product. Employing next generation sequencing (NGS) tools, such as 16s RNA gene based microbiome sequencing could allow researchers to gain further insight into the microbial populations present through various niches in processing.

In this study, 16S high throughput sequencing (HTS) was utilized to establish a typical microbiome of commercially processed broilers. Furthermore, establishing NGS as an applicable tool, in conjunction with currently available plating techniques, was done to validate and measure the reduction in microorganisms by the antimicrobial multi-hurdle system of commercial processors. Lastly, this study evaluated the microbiome profile to identify potential indicator organisms that could benefit the broiler industry during bio-mapping.

## Materials and Methods

### Sample Collection

**FIGURE 1:**
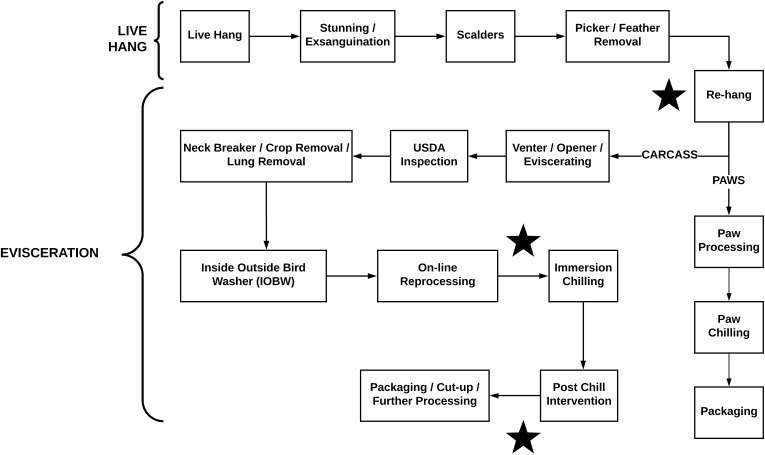
Diagram of the broiler slaughter process. The stars represent sampling locations.

Whole chicken carcass rinsates were collected from three commercial broiler abattoirs. The birds were aseptically removed from the production line shackles during 1st shift production; each location had been processing for a minimum of 3 h prior to sampling. A total of 30 rinsates were collected at each slaughter facility and each facility had 3 sampling points (**Figure 1**) defined as rehang, pre-chill, or post-chill. In all, 90 carcasses were aseptically collected from the processing line and rinsed in pre-chilled 400 mL Butterfield’s Phosphate diluent as prescribed in the Microbiological Laboratory Guidebook (MLG) Food Safety and Inspection Service USDA (2017). The rinsates was placed back into the original Butterfield’s Phosphate diluent container with screw lids sealed. They were placed on ice for transport and returned to the testing lab for analysis. Upon arrival the samples were placed into the refrigerator.

### Bacterial Enumeration

All samples were plated as described by the USDA MLG Chapters 3 and 41.5 on following media: 3M Aerobic Plate Count (APC) PetriFilm and 3M *Enterobacteriaceae* PetriFilm (3M Microbiology, Saint Paul, MN, United States). Prior to enumeration, samples were re-suspended in their respective jars by shaking vigorously and subsequently performing 1:10 serial dilutions in Butterfield’s Diluent (BF’s) (Edge Biologicals Inc., Memphis, TN, United States). One milliliter aliquots were directly plated from the sample and dilution tubes onto the corresponding PetriFilm. PetriFilm plates were incubated at 35°C in aerobic conditions per the manufacturer’s directions. Samples were incubated per the manufacturer’s directions and colonies were enumerated and calculated as total colony forming units (CFU) per mL for each dilution.

### DNA Extraction

A 50 mL subsample of the original 400 mL WBCR was transferred into a sterile 60 mL conical tube. The conical tubes were spun down using an Thermo Scientific Sorvall Lynx 6000 (Langenselbold, Germany) at 8,000 × *g* for 15 min. The supernatants were poured off and the pellets were subsequently re-suspended in 2 mL of phosphate buffered saline (PBS). DNA extractions were performed using a Fisher Scientific AccuSpin Micro 17 (Langenselbold, Germany) and a QIAamp DNA Stool Mini Kit (Qiagen, Valencia, CA, United States) with modifications to increase DNA yield (Park et al., 2014, 2016). The specific modifications were performed prior to the QIAamp Stool Mini Kit (Qiagen, Valencia, CA, United States) and included the addition of 0.7 mm garnet beads (MO BIO Laboratories Inc., Carlsbad, CA, United States) and vortexing for 1 min. The samples were centrifuged and the supernatant was transferred to a fresh 2 mL microcentrifuge tube containing 0.1 mm glass beads (MO BIO Laboratories Inc., Carlsbad, CA, United States). Those tubes underwent horizontal vortexing for 10 min. and then incubated in a 95°C heat block for 6 min (Park et al., 2014). QIAamp DNA Stool Mini Kit was performed as prescribed by the manufacturer. All samples were analyzed on a Qubit^®^ 2.0 Fluorometer (Life Technologies, Carlsbad, CA, United States) to determine the isolated DNA concentration followed by dilution to 10 ng/μL.

### Library Preparation

The isolated DNA aliquots were utilized to construct a sequencing library that targeted the V4 region of 16S rRNA as suggested by Kozich et al. (2013). Individual DNA samples were amplified with dual-index primers through PCR and amplicons were normalized using the SequalPrep^TM^ Normalization Kit (Life Technologies) per the manufacturer’s recommendation. Each sample contained unique barcode sequences, at both the front and end of the PCR amplicon, to distinguish each sample sequence in a pooled library. The pooled library contained a 5 μL aliquot of each normalized sample and was used for further assays. Once pooled, the library concentration and the exact DNA product size were measured using a KAPA Library Quantification Kit (Kapa Biosystems, Woburn, MA, United States) through quantitative PCR (qPCR, Eppendorf, Westbury, NY, United States) assay and an Agilent 2100 Bioanalyzer System (Agilent, Santa Clara, CA, United States), respectively. Based on the qPCR and bioanalyzer results, the pooled library was subsequently diluted to 4 nM prior to sequencing.

### Sequencing via an Illumina MiSeq Platform 157

A pooled library (20 nM) and a PhiX control v3 (20 nM) (Illumina) were mixed with 0.2 N fresh NaOH and HT1 buffer (Illumina) to produce the final concentration of 12 pM’s each. The resulting library was mixed with the PhiX control v3 (5%, v/v) (Illumina) and 600 μL loaded on a MiSeq^®^ v2 (500 cycle) Reagent cartridge for sequencing. All sequencing procedures were monitored through the Illumina BaseSpace^®^ website.

### Sequencing Data Processing

Both demultiplexed R1 and R2 sequencing read (approximately 250 bp in length) files were acquired from the Illumina BaseSpace^®^ website and data processing was performed using the QIIME pipeline (version 1.9.1) (Caporaso et al., 2010; Park et al., 2016). Clustered sequences were used to assemble Operational Taxonomic Units (OTUs) tables with 93.93% identity and classified into the respective taxonomical level from domain to genus based on the Greengenes 16s rRNA gene database. Within the QIIME 1.9.1 package, both alpha diversity and beta diversity data were obtained. Alpha diversity data included rarefaction curves for OTUs and Chao1, while beta diversity data included weighted and unweighted UniFrac distances to characterize the microbial population.

### Statistical Analysis

All bacterial counts were log 10 transformed, prior to analyzing the mean and standard deviation of each individual plant. A One-way Analysis of Variance (ANOVA) or Tukey’s Honest Significant Difference Test was performed using JMP^®^ (version 13.1.0). Microbiome alpha and beta diversity were calculated by using QIIME pipeline (version 1.9.1). Additionally, quality metrics from the Illumina Mi-seq runs were obtained from Illumina BaseSpace^®^ website.

## Results

### Quantitative Bio-Mapping Results

**FIGURE 2:**
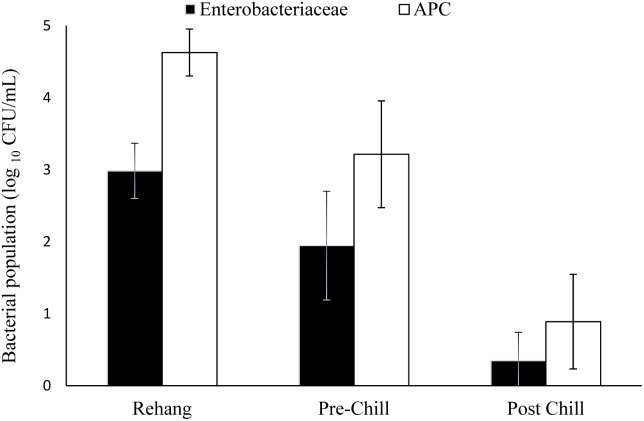
Bio-map of evisceration. The bacterial mean log CFU/mL counts for both *Enterobacteriaceae* and aerobic plate counts (APC).

The log means of individual and all evisceration microbial data for both APC and *Enterobacteriaceae* are presented in **Table 1**. The microbial reduction from rehang to post-chill did have a statistically significant reduction, *p*-value < 0.001, as indicated by **Table 2**. The APC all plant mean log CFU/mL bacterial counts for rehang, pre-chill, and post-chill were 4.63, 3.15, and 0.81, respectively; *Enterobacteriaceae* was 2.99, 1.79, and 0.12, respectively. This data was utilized to build the bio-map in **Figure 2**. The reduction from rehang to post-chill for APC was 3.82 log CFU/mL and *Enterobacteriaceae* was 2.86 log CFU/mL. Each step reduced the microbial populations significantly and **Figure 2** illustrates the reduction throughout the evisceration process. In summary, bacterial counts continued to drop significantly from rehang to post-chill which yielded a negative slope, indicative of a processing system in control.

**Table 1:** Microbial log CFU/mL reduction on Whole Bird Carcasses Rinses.

Step	Plant A		Plant B		Plant C		All Plant	
				
	APC Mean	EB Mean	APC Mean	EB Mean	APC Mean	EB Mean	APC Mean	EB Mean
Rehang	4.92 ± 0.28 a^∗^	3.37 ± 0.24 a	4.52 ± 0.20 a	2.64 ± 0.28 a	4.45 ± 0.29 a	2.94 ± 0.20 a	4.63 ± 0.33 a	2.99 ± 0.38 a
Pre-chill	3.94 ± 0.50 b	2.65 ± 0.44 b	2.83 ± 0.62 b	1.14 ± 0.94 b	2.69 ± 0.12 b	1.59 ± 0.26 b	3.15 ± 0.73 b	1.79 ± 0.88 b
Post-chill	1.12 ± 0.96 c	0.19 ± 0.42 c	0.90 ± 0.51 c	0.16 ± 0.29 c	0.40 ± 0.36 c	0.00 ± 0.00 c	0.81 ± 0.71 c	0.12 ± 0.30 c


*^∗^For each individual plant, testing locations that do not share a similar letter designation are significantly different with a p-value < 0.01.*

**Table 2:** Tukey-Kramer HSD for all plant microbial counts log CFU/mL.

Tukey-Kramer HSD		APC		Enterobacteriaceae	
		
Level	- Level	Difference	*p*-value	Difference	*p*-value
Rehang	Post-chill	3.82	<0.001	2.86	<0.001
Pre-chill	Post-chill	2.34	<0.001	1.67	<0.001
Rehang	Pre-chill	1.47	<0.001	1.19	<0.001


### Taxonomic Summary

**FIGURE 3:**
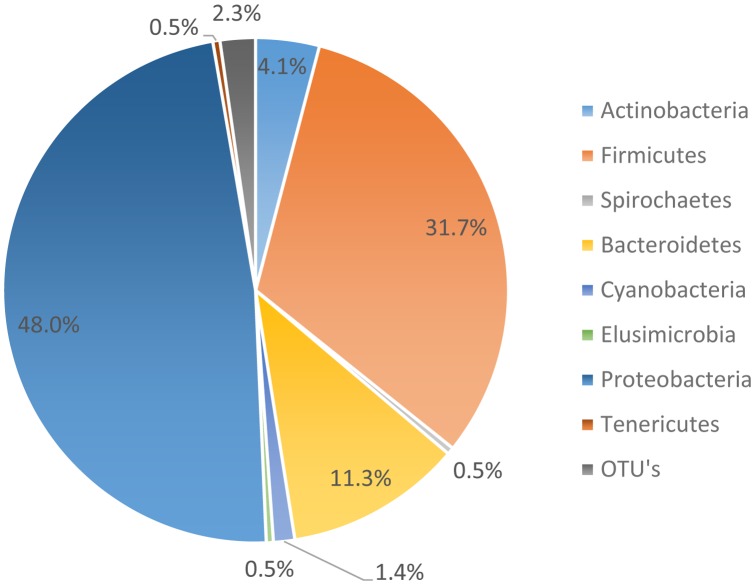
All phylum present through rehang, pre-chill, and post-Chill.

The microbiome data suggests that 95.01% of the organisms present were identified as organisms from the phyla Bacteroides, Firmicutes, Proteobacteria, and Actinobacteria. However, the most abundant phyla, as noted in **Figure 3**, was Proteobacteria. Proteobacteria represented 48.0% of all genomes recovered, followed by Firmicutes with 31.7%, and Bacteroidetes with 11.3%. During the genome analysis of all the rinsates collected at the genus level, a total of 222 OTU’s were identified and only 23 OTU’s or 9.65% was recovered after post-chill.

**FIGURE 4:**
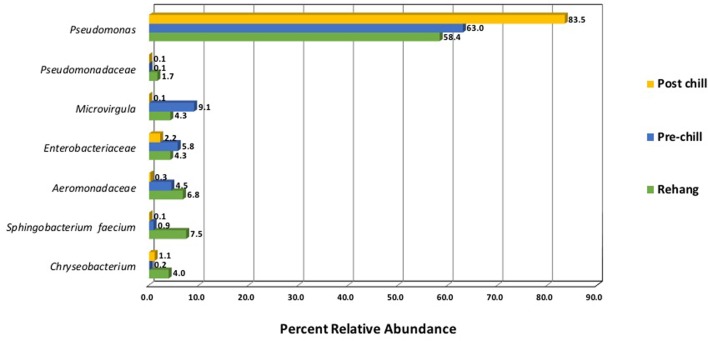
Bio-map of microorganisms through evisceration when the genera were identified at all sampling locations and abattoirs.

Since one objective was to investigate non-pathogenic indicator candidate organisms, the ideal organism would be present at rehang, pre-chill, and post-chill. Therefore, the genera were first filtered by those observed in the post-chill samples only. Therefore, **Table 3** contains a list of all genera recovered at all three post-chill abattoirs. The list of organisms was further filtered by requiring all organisms to be present in rehang, pre-chill, and post-chill samples. Therefore, **Figure 4** indicates genera identified during all sampling stages and abattoirs for a total of 7 OTU’s at the taxonomic level Family or Genus. The two taxonomic groups with the highest relative abundance were *Pseudomonas* and *Enterobacteriaceae*. The post-chill relative abundance of *Pseudomonas* and *Enterobacteriaceae* was 83.5 and 2.2%, respectively. Identified genera with a relative abundance >1.0% were analyzed at the species level (**Table 4**). Few species at post-chill were identified (**Table 4**) and those identified were <1.0%. The OTU’s most abundant were closely related to *Pseudomonas*, *Enterobacteriaceae*, and *Chryseobacterium* with a relative abundance of 94.8, 2.2, and 1.13%.

**Table 3:** All identified microorganisms present at post-chill and processing abattoirs.

OTU ID	All plant mean % abundance
*Pseudomonas*	83.51
*Enterobacteriaceae*	2.23
*Bacteroides*	1.46
*Chryseobacterium*	1.13
*Flavobacterium*	0.37
*Moraxellaceae*	0.36
*Aeromonadaceae*	0.30
*Ruminococcaceae*	0.21
*Clostridium*	0.20
*Mycoplana*	0.14
*Psychrobacter*	0.14
*Oxalobacteraceae*	0.13
*Acinetobacter*	0.12
*Sphingobacterium*	0.10
*Microvirgula*	0.06
*Pseudomonadaceae*	0.06
*Paenibacillus*	0.04
*Comamonadaceae*	0.03
*Lachnospiraceae*	0.03
*Clostridiaceae*	0.02
Gammaproteobacteria other	0.02
*Clostridiaceae* other	0.01
*Pelosinus*	0.01


**Table 4:** List of microorganisms present during all testing locations and abattoirs are in bold.

#OTU ID	Rehang % abundance	Pre-chill % abundance	Post-chill % abundance
***Enterobacteriaceae***	4.3	5.8	2.2
*Citrobacter*	0.0	0.0	0.0
*Erwinia*	0.6	0.0	
*Enterobacteriaceae* Other	0.1	0.1	0.0
*Serratia*	0.0	0.0	
*Yersinia*	0.0	0.0	
***Pseudomonadaceae***	1.7	0.1	0.1
*Pseudomonadaceae* Other	0.0	0.0	0.0
***Pseudomonas***	45.9	60.6	71.7
*Pseudomonas fragi*	0.0		0.0
*Pseudomonas* Other	10.7	1.9	23.0
*Pseudomonas veronii*	0.5	0.2	0.3
*Pseudomonas viridiflava*	1.3	0.3	0.1
***Chryseobacterium***	4.0	0.2	1.1
***Sphingobacterium faecium***	7.5	0.9	0.1
***Aeromonadaceae***	6.8	4.5	0.3
***Microvirgula***	4.3	9.1	0.1


**Species identified when the genera were present in >1.0% relative abundance at post-chill.**

### QIIME Sequencing Metrics

**FIGURE 5:**
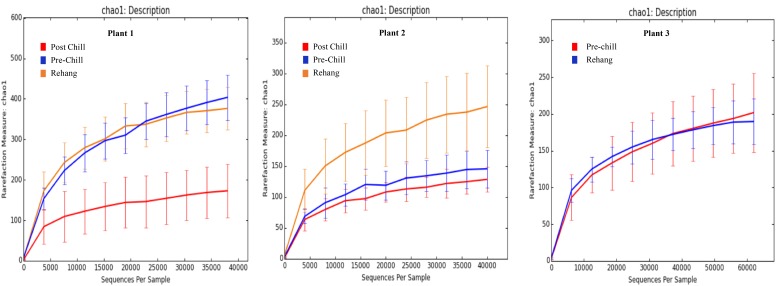
Chao 1 rarefaction curve. The measure of richness within a community at each processing abattoir and testing location within the plant.

**FIGURE 6:**
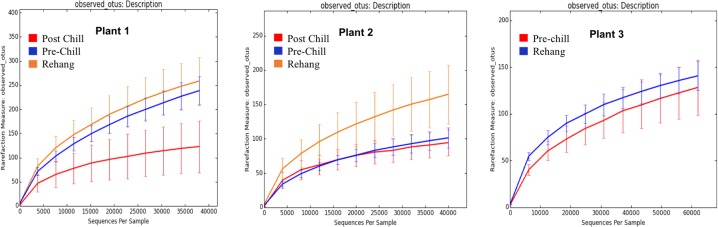
Operational Taxonomic Unit (OTU) rarefaction curves. The number of observed OTU’s versus the length of sequence read at each processing plant and testing location within the plant.

During sequencing, 18,879,978 reads were generated and 17,730,162 of those reads passed filtering. Therefore, 93.93 ± 0.53% of the sequence clusters passed filtration with an error rate of 1.75 percent. Additionally, BaseSpace reported 82.1% of base calls having a Q30 score or better; a quality metric indicating that 1 in 1000 base calls have a possible error. The summarized Illumina Mi-Seq read lengths and Shannon Diversity values obtained from QIIME are identified in **Table 5**. The standard deviations associated with Shannon diversity scores were obtained using JMP. As expected, the samples exhibited a more diverse population in the less processed rehang rinsates and as the carcasses were further processed they become less diverse. Additional alpha diversity results are from Chao1 and OTU’s rarefaction curves presented in **Figures 5**, **6**. Both **Figures 5**, **6** indicate the read lengths and the number of organisms’ present for the associated sample location. These curves indicate that the diversity within the sample were higher during rehang and became less diverse by the end of post-chill. The loss in community richness should be expected as the carcasses are undergoing cleaning steps and does resemble the finding obtained in the bio-map.

**Table 5:** Summary of Illumina Mi-Seq read lengths and QIIME Shannon Diversity.

Alpha diversity				

Sample location		Read length	Shannon	*SD*
Plant 1	Rehang	38000	2.98	0.51
	Pre-Chill	38000	2.94	0.42
	Post-Chill	38000	0.85	0.64
Plant 2	Rehang	40000	2.52	0.54
	Pre-Chill	40000	2.05	1.56
	Post-Chill	40000	1.50	2.19
Plant 3	Rehang	62000	1.90	0.30
	Pre-Chill	62000	1.27	0.54
	Post-Chill	62000	NA	NA


**FIGURE 7:**
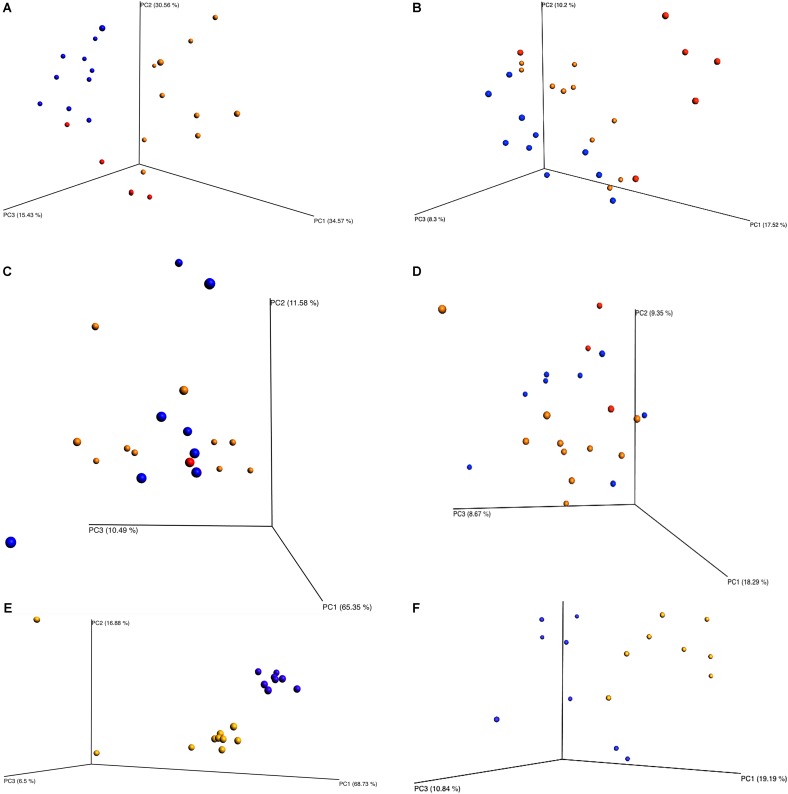
Beta diversity between sampling locations and individual processing abattoir. Weighted and unweighted UniFrac PCoA plots **(A)** Plant 1 weighted. **(B)** Plant 1 unweighted. **(C)** Plant 2 weighted. **(D)** Plant 2 unweighted. **(E)** Plant 3 weighted. **(F)** Plant 3 unweighted. Orange is for rehang, blue is for pre-chill, and red is for post-chill.

**FIGURE 8:**
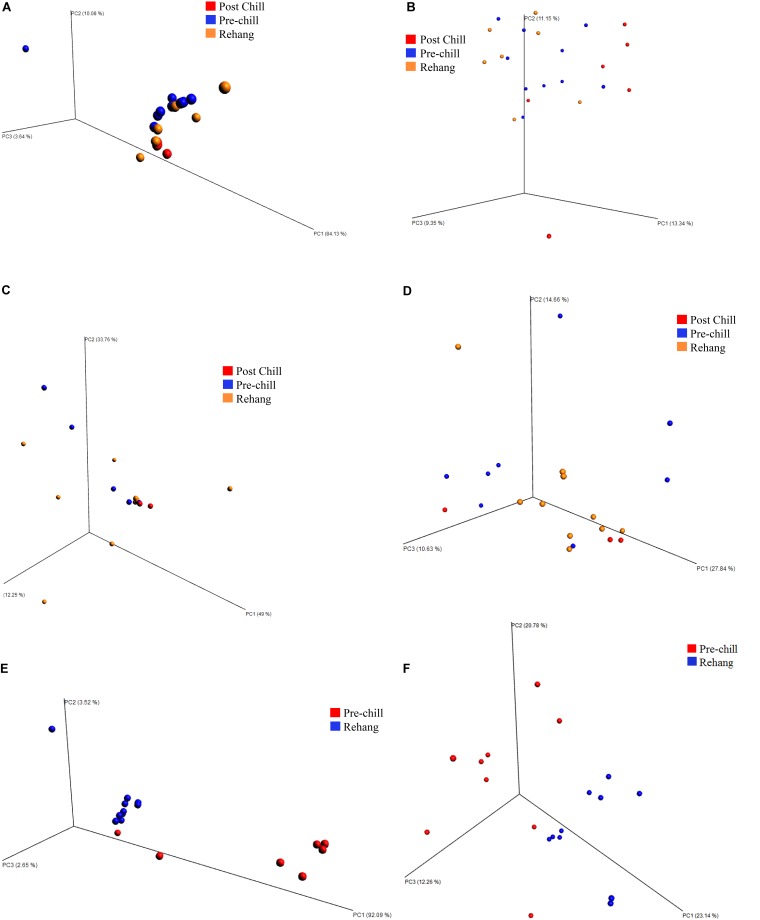
Beta diversity among *Pseudomonas* spp. between sampling locations and individual processing abattoir. Weighted and unweighted UniFrac PCoA plots **(A)** Plant 1 weighted. **(B)** Plant 1 unweighted. **(C)** Plant 2 weighted. **(D)** Plant 2 unweighted. **(E)** Plant 3 weighted. **(F)** Plant 3 unweighted. Orange is for rehang, blue is for pre-chill, and red is for post-chill.

Beta diversity principle coordinate analyses, **Figure 7**, depicted the relatedness of identified OTU’s between samples. Both weighted and unweighted UniFrac plots (**Figure 7**) were generated for plants 1, 2, and 3. The weighted PCoA UniFrac plot quantitatively measured the relative abundance of OTU’s among a group. The unweighted PCoA UniFrac plot was a qualitative representation of phylogenetic distance based on the presence/absence of OTU’s among samples in a group. Initial analysis of the PCoA plots for all organisms present indicated less genetic diversity among the total population of young broilers (**Figure 7E**). As the birds increased in age the population grew in genetic diversity (**Figure 7A**). However, the inverse was true for the PCoA plots generated for *Pseudomonas* (**Figure 8**). Rather, the PCoA plots in **Figure 8** indicate that broilers with an older slaughter age had greater similarity in genetic diversity for the population of *Pseudomonas* spp. Since *Pseudomonas* spp. had the highest relative abundance in all samples and locations collected, **Figure 8** depicts weighted and unweighted PCoA plots generated for *Pseudomonas* spp. only. **Figures 8A,C,E** are the weighted PCoA plots for Plants 1, 2, and 3. These figures depict shifts in the relative abundance in *Pseudomonas* spp. as the birds increase in slaughter age, where Plant 1 (**Figure 8A**) is the oldest and Plant 3 (**Figure 8E**) is the youngest. As for the unweighted PCoA plots, **Figures 8B,D,F**, indicate a greater phylogenetic difference for *Pseudomonas* spp. in **Figure 8F** and an increase in similarity in **Figure 8B**.

## Discussion

The quantitative data obtained in this investigation demonstrate the successful reduction of the bacterial load during the stages of evisceration. The data was utilized to build a biological map of the process and the additional microbiome profiles provided further insight into the organisms that were most prevalent through the evisceration process. In previous research, Zhang et al. (2011) reported post-chill results with an APC mean log 1.79 CFU/mL. Additionally, APC and *Enterobacteriaceae* exhibited post-chill results of mean log 2.86 and 0.66 CFU/mL, respectively (Handley et al., 2010). An investigation on the effectiveness of chlorinated chill water, James et al. (1992) reported post-chill carcasses yielding mean log 2.51 CFU/mL for APC and mean log 1.75 CFU/mL for *Enterobacteriaceae*. Similarly, Berrang and Dickens (2000) noted APC pre-chill and post-chill carcass counts of mean log 3.6 CFU/mL and 2.9 CFU/mL. As for pre-chill, Bauermeister et al. (2008) recovered APC mean log CFU/mL 4.24 from commercially processed carcasses.

Interestingly, the microbial counts obtained over these last 20 years have decreased as expected per changes in processing intervention strategies. Alternative antimicrobial strategies have been extensively investigated and are currently approved for the USDA Safe and Suitable List, such as formic acid, citric acid, lactic acid, propionic acid, peroxyacetic acid, tri-sodium phosphate, chlorine dioxide, acidified sodium chlorite, and cetylpyridinium chloride (Mulder et al., 1987; Izat et al., 1990; Dickens et al., 1994; Lillard, 1994; Doyle and Waldroup, 1996; Yang et al., 1998; Ricke, 2003; Ricke et al., 2005; Bauermeister et al., 2008; Sofos et al., 2013; Kim et al., 2017). These research studies and reviews have provided evidence that each antimicrobial has an optimal mode of application, such as dips, rinses, sprays, or chill tank use. Additionally, each intervention can be more effective on certain bacterial groups than others. For instance, it has been noted previously that citric acid was more effective against Gram positive bacteria than Gram negative bacteria (del Rio et al., 2007a,b; Alonso-Hernando et al., 2009). Hunter et al. (2009) reported a reduction in the *Campylobacter* subspecies diversity from rehang to post-chill using NGS.

More recently, microbiome analyses have been performed on the following poultry matrices, fecal, litter, carcasses, carcass weeps, and chlorinated chill tank water (Oakley et al., 2013; Rothrock et al., 2016; Kim et al., 2017). These studies noted shifts in the microbiota through the production process and noted within this study. The multiple interventions in the slaughter and evisceration process reduced both the microbial load and the diversity of the microbiome. Kim et al. (2017) observed a similar sample profile as this study, where 98.7% of the phyla present were identified as Firmicutes, Proteobacteria, Bacteroidetes, Actinobacteria, and Cyanobacteria. The organisms present in this study have also been previously reported by other researchers analyzing meat sample microbiomes or from meat spoilage investigations (Borch et al., 1996; Patsias et al., 2006; Nychas et al., 2008; Handley et al., 2010; Rothrock et al., 2016; Kim et al., 2017). The presence of *Pseudomonas* in fresh carcasses is consistent with observations made by Hanning et al. (2009) when they used PCR to detect and differentiate *Pseudomonas* spp. from retail poultry carcasses. Additionally, *Pseudomonas* spp. have been found to differ between fresh versus refrigerated poultry meat (Arnaut-Rollier et al., 1999; Morales et al., 2016). In characterizing *Pseudomonas* recovered from spoiled poultry fillets, Morales et al. (2016) observed considerable genotypic and phenotypic variability between and within species. Given the predominance of *Pseudomonas* observed in the current study and the genetic variability reported by Morales et al. (2016), whole genome sequencing of *Pseudomonas* spp. throughout processing and cold storage may reveal a pattern of particular strain succession during processing and cold storage. Likewise, the appearance of a particular strain at certain phases of processing may be indicative of the types of antimicrobials being employed. Finally, particular strains could be predictive indicators for increased likelihood of biofilm formation and/or favoring survival of certain foodborne pathogens such as *Campylobacter* (Hanning et al., 2009; Hilbert et al., 2010; Morales et al., 2016).

## Conclusion

The evisceration process largely impacted the microbial diversity on carcass quality. This study identified the potential use of NGS in association with quantitative microbial data to determine the efficacy of a commercial antimicrobial multi-hurdle system. Additionally, broiler carcasses were characterized to establish a typical commercial microbiome profile. As for the identification of potential indicator organisms, *Pseudomonas*, *Enterobacteriaceae*, and *Weeksellaceae Chryseobacterium* were identified as potential indicator organisms because they were isolated from all processing abattoirs and sampling locations.

## Data Availability Statement

Data are available in the following link: https://figshare.com/s/83a369e639f0a54bd77e.

## Author Contributions

JH, SP, SK, and SR designed the experiments. JH performed the experiments and JH, SP, and SK analyzed the data. JH, SP, SK, and SR revised the manuscript.

## Conflict of Interest Statement

The authors declare that the research was conducted in the absence of any commercial or financial relationships that could be construed as a potential conflict of interest.
